# Recent advances of engineered oncolytic viruses-based combination therapy for liver cancer

**DOI:** 10.1186/s12967-023-04817-w

**Published:** 2024-01-02

**Authors:** Junhe Zhang, Yunxi Xiao, Jie Zhang, Yun Yang, Liao Zhang, Fan Liang

**Affiliations:** 1 Institutes of Health Central Plains, Xinxiang Medical University, No. 601 Jinsui Road, Xinxiang, 453003 Henan Province China; 2 Henan Key Laboratory of Neurorestoratology, The First Affiliated Hospital of Xinxiang Medical University, Weihui, 453100 China; 3 School of Basic Medical Sciences, Xinxiang Medical University, Xinxiang, 453003 China

**Keywords:** Gene therapy, Liver cancer, Oncolytic virus, Targeted therapy

## Abstract

Liver cancer is a major malignant tumor, which seriously threatens human health and increases the economic burden on patients. At present, gene therapy has been comprehensively studied as an excellent therapeutic measure in liver cancer treatment. Oncolytic virus (OV) is a kind of virus that can specifically infect and kill tumor cells. After being modified by genetic engineering, the specificity of OV infection to tumor cells is increased, and its influence on normal cells is reduced. To date, OV has shown its effectiveness and safety in experimental and clinical studies on a variety of tumors. Thus, this review primarily introduces the current status of different genetically engineered OVs used in gene therapy for liver cancer, focuses on the application of OVs and different target genes for current liver cancer therapy, and identifies the problems encountered in OVs-based combination therapy and the corresponding solutions, which will provide new insights into the treatment of liver cancer.

## Introduction

Liver cancer is the third leading cause of cancer death worldwide in 2020, with approximately 830,000 deaths [1]. At present, liver cancer is primarily treated with surgical resection and liver transplantation, supplemented by radiotherapy, chemotherapy, targeted therapy and immunotherapy. Although liver resection and liver transplantation are potentially curative, patients must be diagnosed at the earlier stage. Even with strict monitoring, a number of patients with liver cancer are still found in the middle and late stages of the tumor, missing the best time for surgery and liver transplantation, which will often cause ineffective treatment [2]. In addition, liver resection and transplantation still face severe damage to liver function and a shortage of liver donors [3]. The lack of specificity of radiotherapy and chemotherapy to tumor cells has extensive side effects. Gene therapy is a biological therapy in which vectors introduce exogenous genes into target cells to alter gene expression, and diseases are corrected or compensated on the basis of genetic defects and abnormalities [4]. Tumor gene therapy still accounts for the majority of global clinical trials, which has achieved major breakthroughs with the advancement of biotechnology. In 1990, gene therapy for adenosine deaminase deficiency was successful. Since then, many clinical programs have been launched worldwide. Gene therapy depends on appropriate gene delivery vectors because deoxyribonucleic acid and small interfering RNA (siRNA) can be easily degraded in vivo, and they must remain stable in host cells to exert therapeutic effects, which require effective transmembrane delivery vectors [5]. Selecting a suitable delivery vector will enable nucleic acid molecules to reach their action sites, improve delivery efficiency, exert anti-cancer effects, and reduce damage to normal tissues and organs [6]. The commonly used delivery vectors can be divided into two categories: viral and non-viral vectors. Viral vectors have high transfection efficiency, and they are widely used, among which oncolytic virus (OV) vectors are more prominent in gene therapy.

OVs can identify, infect, and dissolve different cells in the tumor environment, indicating its natural tendency to affect tumor cells, and its tumor-specific replication may be an inherent feature of a certain virus. It can specifically infect tumor cells and replicate in these cells by implementing OV genetic engineering. Tumor cells are directly killed by viral infection and dissolution, releasing more virus particles to infect neighboring cells and distant metastases. Viral infection can also activate the immune system to recognize and attack tumors [7]. OVs can selectively infect tumor cells/stromal cells and induce oncolysis, usually in the form of immunogenic cell death that would present danger signals including damage associated molecular patterns (DAMPs), and tumor-associated antigens including neoantigens, further elicit T-cells-mediated adaptive antitumor immunity. In the early twentieth century, there were reports of using viruses to treat tumor patients, but the results of these studies were primarily negative. In some immunosuppressed patients, the virus lacks specificity, and many patients die from viral infection of normal tissues. Until the 1990s, the era of viral genetic engineering to enhance its oncolytic potential has begun. In 1991, a herpes simplex virus (HSV) with negative thymidine kinase and weakened neurotoxicity showed activity in the mouse glioblastoma model [8]. To date, many ongoing or completed trials have used OVs from different viral families, and new OVs continue to enter the clinical stage. In 2015, talimogene laherparepvec was approved by Food and Drug Administration (FDA) for the treatment of advanced melanoma [9, 10]. In brief, through decades of research, the mechanism of OV replication is gradually clarified, which has been proven to be effective in killing tumors, thereby leading to the direct lysis of tumor cells and stimulating tumor immune response. Therefore, OV is widely used in tumor gene therapy [11]. Table 1 shows the current research status of OV trials used for clinical treatment of various tumors (as of November 30, 2023), as shown in the ClinicalTrilas.gov website. Several researchers have focused on certain OV types and their targeting mechanisms on various tumors [12–15]. To date, there are few reports about the therapeutic modes and strategies of commonly used OVs in liver cancer, such as JX594, OBP301 and VG161 [16–18]. Table 2 shows some oncolytic virus products in clinical trials for liver cancer. Here, we comprehensively discuss the current status of OVs and different target genes used for liver cancer therapy, and briefly cover the combination gene therapy strategies using OVs for liver cancer.

**Table 1:** Current research status of oncolytic viruses

Virus	Types of cancer	Research institution	Experimental phase	NCT number
HSV	Melanoma	Bristol-Myers Squibb/Takara Bio Inc	Phase II	NCT01017185
	Solid tumor	Wuhan Binhui Biotechnology Co. Ltd	Phase I	NCT04386967
	Gastrointestinal cancer	Wuhan Binhui Biotechnology Co. Ltd	Phase II	NCT05648006
	Pancreatic cancer	Wuhan Binhui Biotechnology Co. Ltd	Phase I/II	NCT04637698
	High-grade glioma	University of Alabama at Birmingham	Phase II	NCT05632562
	Solid tumor	Takara Bio Inc	Phase I	NCT01017185
NDV	Metastatic cancer	Hadassah Medical Organization	Phase II	NCT00348842
Ad	Recurrent glioblastoma	National Cancer Institute (NCI)	Phase I	NCT00004080
	Pancreatic cancer	Lokon Pharma AB	Phase I/II	NCT02705196
	Primary peritoneal cancer	University of Alabama at Birmingham	Phase I	NCT00562003
	Solid tumor	TILT Biotherapeutics Ltd	Phase I	NCT04695327
	Liver cancer	Emergent BioSolutions	Phase I	NCT03160339
	Recurrent glioblastomaLung	Clinica Universidad de Navarra	Phase I	NCT03714334
	Cancer (NSCLC)	Benjamin Movsas, M.D	Phase I	NCT03029871
	Ovarian cancer	TILT Biotherapeutics Ltd	Phase I	NCT05271318
	Advanced/metastatic solid tumours	Canadian Cancer Trials Group	Phase I	NCT02285816
VSV	Endometrial cancer	Mayo Clinic	Phase I	NCT03120624
	Advanced malignant solid neoplasm	Mayo Clinic	Phase I	NCT01628640
	Hepatocellular carcinoma	Mayo Clinic	Phase I	NCT01628640
Reovirus	Recurrent melanoma	National Cancer Institute (NCI)	Phase I	NCT00651157
	Unspecified childhood solid tumor	National Cancer Institute (NCI)	Phase II	NCT01240538
	Recurrent primary peritoneal carcinoma	National Cancer Institute (NCI)	Phase II	NCT00602277
	Pancreatic acinar cell carcinoma	National Cancer Institute (NCI)	Phase II	NCT01280058
	Metastatic melanoma	National Cancer Institute (NCI)	Phase II	NCT00651157
	Metastatic colorectal cancer	Oncolytics Biotech	Phase I	NCT01274624
MV	Malignant pleural mesothelioma	Mayo Clinic	Phase I	NCT01503177
	Anaplastic astrocytoma	Mayo Clinic/National Cancer Institute (NCI)	Phase I	NCT00390299
	Glioblastoma	Mayo Clinic/National Cancer Institute (NCI)	Phase I	NCT02709226
	Stage IV breast cancer	Mayo Clinic/National Cancer Institute (NCI)	Phase I	NCT04521764
	Recurrent non-small cell Lung cancer	Vyriad, Inc	Phase I	NCT00002625
	Malignant mesothelioma	University of Arkansas	Phase I	NCT01503177
	Metastatic breast cancer	Mayo Clinic	Phase I	NCT01846091
Poxvirus	Advanced tumors	Hangzhou Converd Co., Ltd	Phase I	NCT05914376
	Refractory cancer	National Cancer Institute (NCI)	Phase I/II	NCT02759588
	Malignant solid tumors	Tasly Tianjin Biopharmaceutical Co., Ltd	Phase I/II	NCT04226066
	Hepatic carcinoma	Jennerex Biotherapeutics	Phase II	NCT00554372
	Hepatic carcinoma	Jennerex Biotherapeutics	Phase I	NCT00629759
	Advanced solid tumors	Genelux Corporation	Phase I	NCT00794131
	Renal cell carcinoma	SillaJen, Inc	Phase I/II	NCT03294083

**Table 2:** Some oncolytic virus products in clinical trials for liver cancer

Type of OVs	Product name	Pharmaceuticals company	Route of administration	Clinical phase
AdV	OBP-301	Oncolys Inc	Intratumoral	II
AdV	Onyx-015	Onyx Inc	Intratumoral	II
AdV	SynOV1.1	Beijing SyngenTech	Intratumoral	I
Poxvirus	Pexa-vec	Jennerex Inc	Intratumoral, intravenous	III
Poxvirus	JX594	Jennerex Inc	Intratumoral	II
HSV	SEPREHVIR	Sorrento Inc	Intratumoral	II
HSV	T-vec	Amgen	Intratumoral	II
HSV	MVR-T3011	Invbio	Intratumoral	II
HSV	VG161	Virogin Biotech	Intratumoral	II
HSV	RP2/3	Replimune	Intratumoral	I

## Current situation of OV for gene therapy of liver cancer

Viruses can express their genes with high efficiency in host cells, which makes them suitable as gene delivery vectors for gene therapy and immunotherapy, and the delivery efficiency of viral vectors is high. OVs are the primary therapeutic agents, they destroy tumor cells and induce an antitumor immune response, whereas in replication-deficient systems the tumor toxic gene is the therapeutic agent. Thus, arming OV with tumor toxic genes is a way to enhance the antitumor effects. The OVs optimized by genetic engineering can specifically recognize and infect tumor cells, which have been used in the treatment of various cancers, including liver cancer. The most studied OVs by genetic engineering include HSV, Newcastle disease virus (NDV), measles virus (MV), poxvirus, and adenovirus (AdV). Table 3 shows main genetically engineered virus strains.

**Table 3:** Main genetically engineered virus strains

Oncolytic virus	Viral structures	Critical pathway of specific infections in tumors	Partial genetically engineered oncolytic virus strains	References
Herpes simplex virus	Linear double-stranded DNA virus	Interferon signaling pathway	NV1020, G207, HF-10, HSV-1716, T-VEC, G47∆, M032, RP1, RP2, R36166, DM33, C134	[20, 21, 25]
Newcastle disease virus	Single-stranded RNA virus	Interferon signaling pathway	Lasota, Anhinga, Mukteswar	[28–31]
Vaccinia virus	Linear double-stranded DNA virus	EGFR-Ras signaling pathway	Wyeth, Lister, Copenhagen, Western Reserve, Tian Tan, Modified, Vaccinia Ankar	[41, 42]
Measles virus	Single-stranded RNA virus	Density differences of CD46, SLAM and Nectin-4 receptors	MV-CEA, MV-NIS	[39]
Adenovirus	Linear double-stranded DNA virus	Rb and p53 cell signaling pathway	H101, ONYX-105, DNX-2401, LOAD703, VCN-01, OBP-301, ONCOS-102	[44, 45, 95]

### Herpes simplex virus

Herpes simplex virus is a modified OV because of its rapid infectious properties, broad tropism for different types of tumor cells, minimal mutation of host–cell DNA, and efficacy in accidental HSV infection of medical treatment coverage. At present, HSV-1 and HSV-2 has been identified [19]. HSV-1 is an enveloped double-stranded DNA virus containing a 150 kb genome. Its neurotropic properties, combined with its infectivity and lytic activity, provide ideal characteristics for effective and engineered OVs, which can be used as the vector of tumor gene therapy and the skeleton of OV [20]. Many genetically modified tumors are identified to enhance the specificity of HSV-1 to tumor cells and reduce its autoimmunity and toxicity, including the G207 strain, which has two γ34.5 s containing two loci deletions. γ34.5 can encode an infectious cell protein 34.5 (ICP34.5), indicating its neurotoxicity. In addition, the viral ribonucleotide reductase (vRR) encoded by ICP6 can cause wild-type HSV to replicate even in quiescent cells. Therefore, the replicability of HSV in quiescent cells will be hindered because of the lack of ICP6 gene. HSV has five immediate-early (IE) proteins, among which ICP0 and ICP4 play a key role in activating viral mRNA synthesis and promote the synthesis of early and late proteins. These proteins can replicate and package the HSV genome to form new viral particles. ICP0 can transform the main transcriptional regulator of HSV, ICP4, from a weak transcriptional activator to a powerful mRNA synthesis activator. The combination of ICP0 and ICP4 can promote mRNA synthesis more than ICP0 or ICP4 alone. Moreover, another important IE protein is ICP47. It escapes from host immune response and binds to antigen processing-related transporter (TAP), which reduces the peptide transport function of TAP, effectively inhibits the binding of viral antigen peptide to newly synthesized MHC I molecules, and significantly reduces MHC I expression on the surface of tumor cells, thereby interfering with MHC I-mediated cytotoxic lymphocyte (CTL) activation. G47∆ increases the deletion in the ICP47 region based on G207, enhances viral replication, and increases the presentation of class I molecules of the major histocompatibility complex by tumor cells [20–22]. This genetically engineered HSV-1 has been further studied in liver cancer. For example, genetically engineered HSV-1 mutant Rrp450 does not express vRR, and virus particles of Rrp450 progeny infected with hepatocellular carcinoma (HCC) cells are 3–4 logarithmic orders of magnitude higher than those infected with normal hepatocytes. After intravascular injection, diffused HCC can be significantly reduced [23]. Nakatake et al. confirmed that T-01 has a cytotoxic effect on various human liver cancer cell lines in vitro. Nude mouse models of subcutaneous, orthotopic, and peritoneal xenografts also show an inhibitory effect on tumor growth caused by human hepatoma and hepatoblastoma (HB) cell lines, without damaging the surrounding normal tissues and through T cell-mediated immune response [24]. To date, a modified HSV-1, namely, T-VEC, is the only FDA-approved OV therapy [25]. HSV-2 may be more suitable for oncolysis than HSV-1. oHSV2hGM-CSF is a replication-competent attenuated HSV-2, which is specific for cancer infection, including liver cancer cells, by deleting the viral genes ICP34.5 and ICP47 and inserting the gene-encoding human granulocyte–macrophage colony-stimulating factor (hGM-CSF). Studies on numerous human tumor cell lines have demonstrated that ohHSV2hCM-CSF can effectively engraft the growth of cancer cells and achieve a tumor-suppressive effect [26]. At present, the modified HSV-2 has been used in the phase II clinical trial of melanoma [27], and further research is needed forthe treatment of liver cancer.

### Newcastle disease virus

Newcastle disease virus is an RNA virus belonging to the Paramyxoviridae family. NDV can be divided into three types, namely, virulent, poisonous, and attenuated strains, which primarily infects poultry. Given the defective interferon signaling pathway in cancer cells, NDV can easily infect, replicate, and lyse human cancer cells without affecting normal cells and triggering the innate and acquired immune response [28–30]. Since 1964, Cassel and Garrett [31] first published the anti-tumor effect of NDV, which is a new ideal virus for tumor treatment, and it has been continuously studied and optimized. CD147, also known as an extracellular matrix metalloproteinase inducer, is a highly glycosylated type-I transmembrane protein and an adhesion molecule. The expression level of CD147 is upregulated in many malignant tumors, which can stimulate fibroblasts to produce multiple matrix metalloproteinases (MMPs), whereas MMP-2 and MMP-9 can promote the invasion of liver cancer cells. Therefore, the monoclonal antibody HAb18 against human CD147 is an effective treatment measure for cancer, including liver cancer^32^. Wei et al. constructed a recombinant NDV carrying a chimeric HAb18 antibody (cHAb18), thereby leading to the cHAb18 expression in situ in orthotopic HCC xenografts, enhancing the inhibition of residual tumor cell migration, inducing tumor necrosis, reducing intrahepatic metastasis, and prolonging the survival in mice [15]. Abdullahi et al. proved that the chimeric recombinant vesicular stomatitis virus and NDV vector could rapidly and effectively form syncytium in the HCC cell line, which can prolong the survival time of tumor-bearing mice with HCC in situ; thus, it becomes a new potential vector for the clinical transformation of immunotherapy for HCC [12]. Wu et al. introduced NDV Anhinga strain as a vaccine vector to express IL-2, which proved that the recombinant NDV-expressing IL-2 could enhance the in vivo anti-tumor ability, and it can not only directly kill tumors, but also cause an immune response and solid immune memory in vivo, thereby enhancing the anti-tumor characteristics by increasing the infiltration of lymphocytes in vivo. Thus, this strain may be a powerful candidate for clinical cancer treatment, particularly for liver cancer [33]. Wu et al. also confirmed that the recombinant NDV-expressing tumor necrosis factor-related apoptosis-inducing ligand (TRAIL) could effectively inhibit liver cancer without apparent toxicity [34]. Therefore, more therapeutic genes can reveal their effectiveness in inhibiting liver cancer through NDV and provide important reference for further clinical treatment.

### Measles virus

Measles virus is a paramyxovirus, which can produce highly polymorphic particles, and the tropism of MV primarily depends on the use of its receptor. The Edmonton vaccine strain primarily enters cells through the CD46 receptor, and CD46 is expressed in all nucleated human cells. However, decellularization caused by infection requires a specific receptor density, and CD46 is often overexpressed in many human cancers, which causes MV to preferentially infect tumors and spread [35–37]. At present, the potential application of MV as an innovative cancer treatment method has been studied, which can selectively replicate in cancer cells and kill them, and further activate the anti-tumor immune response. Genetic engineering can also improve tumor specificity and therapeutic effect [38]. Since 1954, Enders and Peeble isolated MV from a patient named Edmonton. They obtained the MV-attenuated Edmonton MV (MV-EDM) vaccine strain through further continuous passage, which can preferentially infect and dissolve various cancer cells and express human carcinoembryonic antigen (MV-CEA) or human sodium iodide transporter (MV-NIS) by genetic engineering to obtain a virus strain with more substantial oncolytic effects [39]. Based on the MV loaded with suicide gene super-cytosine deaminase (SCD), MV-SCD has a vigorous oncolytic activity on HCC in vitro and in vivo, which indicated that suicide gene therapy based on MV is a potential new treatment scheme for HCC to overcome the drug resistance of conventional treatment [40]. In the treatment of HCC with MV vaccine strain MV-Edm improved by adopting CD8^+^NKG2D^+^ cells, MV-Edm-infected HCC can enhance the anti-tumor activity of CD8^+^NKG2D^+^ cells, which provide a novel and clinically relevant strategy for HCC treatment [13].

### Poxvirus

Poxvirus is a giant extracellular enveloped virus (EEV) with a linear double-stranded DNA genome, the central genome region includes highly conserved genes in poxvirus, whereas the terminal region encodes viral factors that regulate immunity or destroy host’s self-defense mechanism. Although poxvirus can infect many kinds of mammalian cells, the factors after entry could determine the tropism of cells and hosts. The replication cycle of poxvirus only occurs in the cytoplasm; thus, the DNA virus has no risk to the host genome. In addition, poxvirus has a high degree of immunogenicity and a potent ability to co-stimulate acquired anti-tumor immunity after replication in tumor tissues. At present, six poxviruses from four different genera have been studied as potential OV, and vaccinia virus (VV) is a typical member of orthopoxvirus, which has been widely studied [41]. VV has inherent tumor targeting, and many characteristics of cancer (blocked apoptotic pathway, cell cycle control disorder, and immune escape) become the best cell conditions for the successful replication of VV. In enhancing the specificity of VV in tumor cells, these genes have been modified to produce different cell lines and improve the selectivity of tumors [42]. For example, in the deletion of viral thymidine tyrosine kinase (TK) and viral growth factor (VGF) genes, TK is a critical enzyme for the DNA synthesis of VV. The VGF is a secretory growth factor homolog that binds to the receptor of endothelial growth factor (EGF), thereby inducing the proliferation of peripheral cells, both of which are expressed in tumor cells, and the deletion of this gene leads to tumor-selective viral replication [42]. Wang et al. confirmed that VV-IL-24 carrying IL-24 gene can inhibit the activity of liver cancer cells, and the combination of tumor-inhibiting luteolin can induce apoptosis of liver cancer cells, which can be used as an effective way of gene therapy for liver cancer [43].

### Adenovirus

Adenovirus is a potential vector for tumor gene therapy because of its unique characteristics, including high infection rate, high load, and lack of insertion mutation. The genome of the commonly used human adenovirus type 5 (Ad5) is approximately 36 kb, with a linear double-stranded DNA molecule, and the two sides of deoxyribonucleic acid are hairpin-like inverted terminal repeats (ITRs). Apart from ITRs, another genetic factor of AdV is the packaging signal, which is necessary for the proper packaging of virus transcripts. The genome of AdV consists of early transcription units (E1A, E1B, E2, E3, and E4) and late transcription units (L1–L5). E1A and E1B regions are subunits of the E1 region, and E1A is the first transcription unit expressed after the AdV chromosome enters the nucleus of infected cells. The E1B gene encodes AdV-mediated gene transfer. The E2 transcription unit encodes a protein involved in viral DNA replication. The E3 region codes various proteins; the E3 protein is indispensable for AdV replication in tissue culture. E4 gene products perform a series of functions, and different proteins play a role in viral DNA replication, viral mRNA transport, and splicing. The capsid of AdV plays an important role in the primary stage of infecting the host [44, 45]. Given the genotoxicity and immunogenicity of viral vectors, the necessary replication genes were eliminated to obtain safe and effective viral vectors. The first-generation AdV removes the regulatory genes E1A and E1B. The second-generation AdV lacks other non-structural genes in the vector (E2/E3/E4). The third-generation AdV, also known as helper-dependent adenoviral vector (Hd-AdV), removes all viral coding sequences, leaving only 5′ and 3′ ITRs in the vector except for the packaging signal. Hence, the vector capacity is large, and the structure of Hd-AdV minimizes the cytotoxicity, prolongs the expression of therapeutic genes, and makes Hd-AdV a potential AdV for gene therapy.

However, considerable studies have shown that replication-deficient adenoviral vectors have many disadvantages. Such vectors can infect not only target tumor cells, but also normal cells, and they lack specificity. With the in-depth study of the structure and gene function of AdV, a novel AdV vector, conditionally replicating adenovirus (CRAd), also known as oncolytic adenovirus (OAd), has been developed by modifying AdV based on the distinct specificity between tumor and normal cells. A commonly used Ad5-based CRAd contains a 24 bp mutation in the E1A gene (E1AD24), and the deletion in E1A prevents the binding of pRB to E1, resulting in the inability of E1AD24 protein to promote viral replication. Another CRAd was constructed by deleting a 55KD gene in the E1B region of AdV. The virus can only replicate in cells that lack functional p53 but cannot survive in normal cells with functional p53. Moreover, the third CRAd can be produced using tissue-specific or cancer cell-specific promoters instead of natural E1A promoters. Common tumor-specific promoters include alpha-fetoprotein (AFP) and prostate-specific antigen. OAd can selectively replicate in tumor cells, resulting in cancer cell lysis and inflammation, and can stimulate immune response and host’s immune response to cancer cells, thereby effectively killing tumor cells while preserving normal cells, which will play an important role in gene therapy [46].

More and more viruses are used as OVs for cancer treatment. The M1 virus selectively kills HCC that lacks zinc finger antiviral protein (ZAP). 69% of liver cancer tissues showed a low expression level of ZAP compared with non-cancerous tissues, and studies have shown that small-molecule anticancer compounds that directly target the valosin-containing protein are effective and safe to treat liver cancer when used in combination with OV M1 [47]. In addition, the tumor selectivity of M1 is related not only to ZAP, but also to the cell membrane receptor MXRA8, which provides a dual biomarker for precision medicine of OV M1 in the treatment of cancer [48]. Recombinant nonpathogenic polio-rhinovirus chimeras (PVSRIPO) recognize the poliovirus receptor (PVR) CD155, which is widely expressed in tumor cells and major components of the tumor microenvironment (TME) in solid tumors. PVSRIPO is important to recurrent patients with grade IV glioblastoma because it has no potential neurotoxicity, and patients receiving PVSRIPO immunotherapy have shown improved survival [49]. However, the application of these OVs to liver cancer still needs further exploration.

## Different strategies of OVs-based gene therapy of liver cancer

**Fig. 1:**
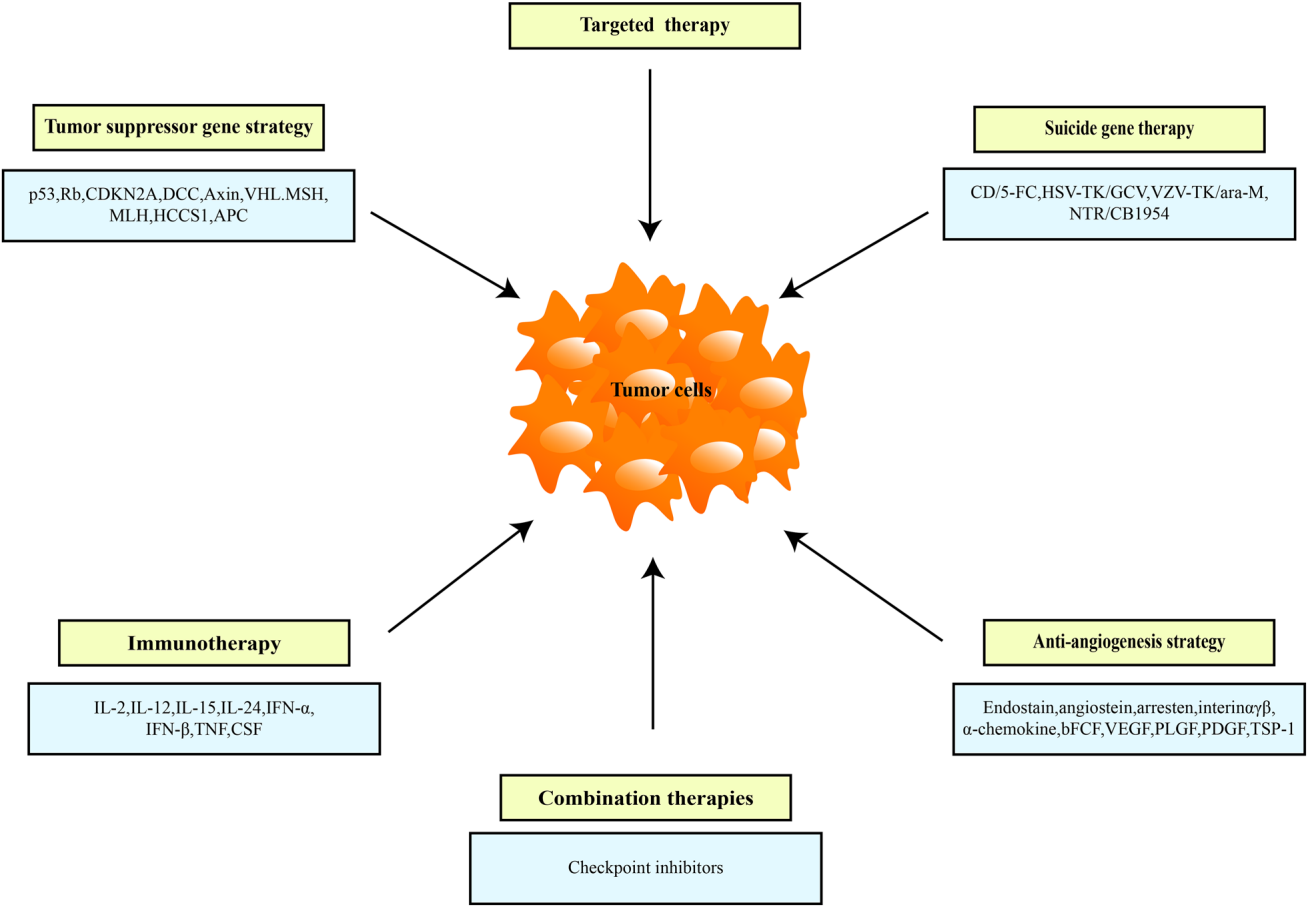
Common gene therapy strategies for the treatment of liver cancer

At present, OV therapy has been widely studied, but most OVs are still in the research stage of laboratory and clinical trials, and their application remains limited because of their shortcomings, such as the complex genome structure of herpes virus. In addition, foreign genes cannot be expressed in host cells for a long time, which can cause immune response, inflammatory response, and systemic toxicity. The structure and biological characteristics of VV is complex, and its safety still needs in-depth study. In recent years, AdVs are the most studied virus in the field of OV therapy because of its relatively simple gene recombination and production, with the most laboratory research and extensive clinical development. With the disclosure of considerable cancer knowledge at genomic and proteomic levels, the number of candidate oncogenes used in gene therapy of liver cancer is increasing. The gene therapy of liver cancer covers a variety of gene transfer strategies that aimed to treat patients with primary and secondary liver cancers, including tumor-suppressor genes, immunotherapy, suicide genes, and anti-angiogenesis. Considerable evidence shows that different gene therapy approaches have synergistic effects when combined with chemotherapy or radiotherapy. Different mechanisms of action may improve these combinations and prevent the development of drug resistance to treatment. The commonly used gene therapy strategies for liver cancer are shown in Fig. 1, and the application of different OVs mediated target genes in liver cancer is shown in Table 4.

**Table 4:** Application of different OVs mediated target genes in liver cancer

Different targeting strategies of gene therapy	Major genetic research targets	Research in gene therapy of liver cancer
Tumor-suppressor gene	Rb, p53, CDKN2A, DCC, Axin, VHL, WTl, MSH, MLH, HCCS1, APC	rAD-p53 has an adequate therapeutic effect on VX2 rabbit liver cancer [52] Ad5-PTEN inhibited the proliferation and migration of HepG2 cells and showed good anti-tumor activity on invasive HepG2 transplanted tumors in nude mice [58] Ad carrying TSLC1 gene inhibits the growth, migration, and invasion of HCC cells by downregulating the Wnt signaling pathway [56]
Immune therapeutic gene	IL2, IL12, IL15, IL-24, IFNα, IFN-γ, IFN-β, TNF, CSF	rAd-IL-2 can stimulate the proliferation of T cells and production of memory T cells in mice with liver cancer and induce tumor-specific CTL reaction and IFN-γ secretion, thereby inhibiting the proliferation of HCC [64] AD-AFP-D55-IL-24 and AD-AFP-D55-TRAIL can induce cell apoptosis, which can significantly inhibit the tumor growth of Huh-7 cell xenograft mice [95]
Suicide gene	CD/5-FC, HSV-TK/GCV, VZV-TK/ara-M, NTR/CB1954	Ad-ETK expressing E1A and HSV-TK can resist HCC in vitro and in vivo, and HSV-TK/GCV enhances OAd therapy [80] AD-VEGFp-CDglyTK can effectively inhibit the growth of HCC cells and vascular endothelial cells in vitro and in vivo [82]
Angiogenesis-related gene	Endostatin, angiostatin, arresten, integrin αγβ3, α-chemokine, bFGF, VEGF, PLGF, PDGF	Ad-DB7-shVEGF can reduce the expression of VEGF in HCC cells and induce an anti-angiogenesis effect in vitro and in vivo [90] ADK1-3 can inhibit the growth of HCC by intravenous injection in mice with HCC [91]

### Tumor-suppressor gene strategy

The essential characteristics of cancer cells include uncontrolled cell proliferation, immortality, genome instability, and the ability to destroy local and distant tissues. The accumulation of DNA mutations causes genomic instability in cancer cells. The balance between the activation and inactivation of tumor-suppressor genes and proto-oncogenes plays a critical role in cancer development. The common mutant tumor-suppressor genes include p53, Rb, and phosphatase and tensin homolog (PTEN) [50]. p53 is the most well studied tumor-suppressor gene in human cancer. Mutations that inhibit the function of p53 are ubiquitous in human cancer, and wild-type p53 has become a critical target gene in tumor gene therapy. In 2003, the first anti-tumor gene therapy drug recombinant human p53 adenovirus (rAd-p53), trade name Gendicine, was approved by China Food and Drug Administration (CFDA) for the combined radiotherapy of head and neck squamous cell carcinoma [51, 52]. Yang et al. compared rAd-p53 combined with fractionated stereotactic radiotherapy (fSRT) for HCC with fSRT alone (fSRT group) or rAd-p53 combined with fSRT (combined group), and the results showed that the combined group was a relatively safe and effective method to treat HCC [53]. Guan et al. conducted a controlled trial of rAd-p53 injection combined with hepatic arterial chemoembolization in the treatment of patients with advanced HCC and those who only received hepatic arterial chemoembolization, and the results showed that the p53 treatment group was significantly better than the control group. Moreover, the patients in the p53 treatment group had fewer gastrointestinal symptoms and better improvement of tumor-related pain. The recombinant human p53 gene has been proven to be a safe and effective treatment for advanced HCC by hepatic arterial chemoembolization [54]. Zhang et al. developed a dual-regulated oncolytic AdV (Ad.wnt-E1A (△24 bp)-TSLC1) that targets Wnt and Rb signaling pathways and carries a tumor-suppressor gene in lung cancer 1 (TSLC1). The results showed that Ad-wnt-E1A(△24 bp)-TSLC1 could effectively lead to autophagic death. The recombinant AdV can effectively induce apoptosis and inhibit metastasis of hepatic cancer stem cell (CSC)-like cells in vivo, further inhibit the growth of transplanted tumor of hepatic CSCs and prolong the survival time of mice [55, 56]. PTEN is a tumor-suppressor gene whose expression is usually lost in tumors, which is highly mutated in many cancers, including HCC. Furthermore, PTEN inhibits PI3K signaling in cell growth on the plasma membrane, while maintaining genomic integrity in the nucleus [57]. The rAd carrying PTEN (Ad5-PTEN) is an effective anti-liver cancer drug; aptamer EpDT3 can specifically bind to epithelial cell adhesion molecule (EpCAM) and target EpCAM-positive cells. The EpDT3-modified Ad5-PTEN gene delivery system can enhance gene expression and cellular uptake in HepG2 cells, inhibit cell proliferation and migration of HepG2, and show superior anti-tumor activity against aggressive HepG2 xenografts in nude mice [58, 59].

### Immunotherapy

Tumor development is related to the relative ability of host immunity. By inducing immunosuppression or inhibiting the relative state of host immunity and changing the expression of marker molecules on the surface of cancer cells, tumor cells can escape the immune recognition of natural killer (NK) cells and other cells; thus, enhancing immune monitoring and suppressing immune escape are important in tumor immunotherapy [60]. As a novel treatment method, immunotherapy has been proven to be effective and safe in treating excessive solid tumors and prolonging median overall survival. Immunotherapy based on antibody and vaccine therapy aims to prevent immunological escape and change immune response [61].

The main immune targets of cancer gene therapy are cytokine/chemokine genes, tumor-related antigens, and fusion proteins, including tumor antigens, genetically modified tumor cells, or immune cells, among which the immune gene therapy of cytokinesis is more prominent [62]. Cytokines have pleiotropic effects, which mediate systemic and local biological effects. Systemic administration of some cytokines, such as IL-2, interferon (IFN), and IL-12, which affect cell growth and differentiation, immune function in several types of cancers. Initial studies have shown that systemic and repeated administration of high doses of cytokines can lead to tumor regression. However, this method is related to incompatible general toxicity. As an anticancer cytokine, IL-2 is secreted by antigen-activated T cells, which can enhance the cytolytic activity of NK cells or lymphokine-activated killer cells [33, 63]. Sun et al. used the rAd expressing IL-2 (rAd-IL-2) in the HCC tumor model, and the results showed that rAd-IL-2 can stimulate the proliferation of T cells and production of memory T cells in mice with HCC, induce tumor-specific cytotoxic T lymphocyte response and increase IFN-γ release, thereby inhibiting the proliferation and development of HCC, which can be an effective method for clinical immunotherapy of HCC [64]. IFN is a pleiotropic cytokine that is different from their sequence identity, the nature and distribution of homologous receptors, and their induced stimulation and source cells. IFN has three main types. Type I interferon mediates many immunomodulatory effects, among which IFN-α induces the production of tumor-specific CTL, and tumor-expressed IFN-α can promote the survival of tumor-specific CTL lines by preventing apoptosis [65]. Studies have confirmed that IFN-α gene therapy induces immunoregulation, anti-proliferation, and apoptosis-promoting activities, which can control tumor growth, reduce the expression of transforming growth factor-β (TGF-β) and tissue inhibitor of metalloproteinase-1 (TIMP-1), and improve liver cirrhosis [66]. KD3 is an Ad formed by the mutation of dl1101/1107 in the E1A region of Ad5. Shashkova et al. confirmed that KD3-IFN constructed by introducing the IFN-α gene to KD3 can inhibit tumor growth in subcutaneous xenografts of Hep3B cells from immunodeficient mice compared with KD3 alone, thereby prolonging survival time and reducing hepatotoxicity [67]. IFN-γ is a cytokine that plays key roles in promoting protective immune response and immunopathology. IFN-γ can inhibit angiogenesis in tumor tissues, induce apoptosis of regulatory T cells, and stimulate the activity of M1 pro-inflammatory macrophages to prevent tumor progression [68].

IFN-α/β and IFN-γ can induce human leukocyte antigen-I (HLA-I) genes[65]. CTLs are the key in gene immunotherapy; in many cases, the activity of CTL has been completely inhibited because of the downregulation of the expression level of HLA-I molecules in HCC cells [69]. Su et al. constructed a human telomerase reverse transcriptase promoter-mediated OAd therapeutic system CNHK300 mIFN-γ, which can induce the degeneration of xenograft in the liver cancer model of immunocompromised and immunocompetent mice through the triple mechanism, including selective tumor dissolution, anti-angiogenesis, and immune response. Moreover, this system has an evident anti-tumor effect, which is of great significance for IFN-γ to be used in gene therapy of liver cancer [14].

Notably, the host immune system protects the body from infections and diseases that can cause damage. In the face of diverse pathogens, mammalian immune systems have evolved complex defense system that produces a large number of antigen receptors to recognize almost all foreign antigens, including pathogens, thereby protecting the host from infection by eliminating pathogens [70, 71]. The host innate immune system constitutes the first line of defense against viruses. During viral infection, viral RNA or DNA is recognized by pattern recognition receptors, and complex signal transduction pathways are initiated to trigger a strong antiviral response, which is then acquired [72, 73]. Therefore, in the treatment of OVs, the viruses are recognized as pathogens by the immune system, and OVs trigger an antiviral immune response in the host, thereby limiting their therapeutic potential, particularly in the treatment of distant tumors[74, 75]. In the environment of anti-tumor and anti-viral immunity, the host triggers the lysis effect of immune system to clear OV infection, thereby decreasing anti-tumor immunity. Therefore, finding a balance between anti-tumor and anti-viral immunity is a problem that must be addressed in OV therapy [75]. For example, the NK cell-mediated OAd delivery system utilizes tumor-homing tropism of NK cells to serve as bioreactors and shelters for the loading, protection, replication, amplification, and release of Ads, which can relieve immunosuppression in the TME [74]. Macrophages are an important part of the host innate immune system, serving as scavenger cells that can recognize and rapidly kill pathogens in a non-specific manner while fighting tumors, thus limiting the efficacy of OVs, and in the treatment of OVs as a double-edged sword, manipulating macrophages to carry viruses into tumors and improve the efficacy of OVs [75].

Various immune cell types in the TME of HCC have been identified as important parameters associated with prognosis and responsiveness to immunotherapy [76]. OVs are considered immunotherapies according to the current state of knowledge, since it is now recognized that their central antitumor effect lies in the activation of a systemic antitumor immune response. As an emerging tumor therapy, OVs preferentially replicate in malignant cells, reverse the immunosuppressive TME, and eventually can be eliminated by the patient. In addition, OVs can modulate the hepatic microenvironment, resulting in a complex interplay between virus and host. The immune system plays a substantial role in the outcome of OV therapy, both as an inhibitor of viral replication, and as a potent mechanism of virus-mediated tumor cell killing.

### Suicide gene therapy

Suicide gene therapy is a potential therapeutic strategy based on introducing a virus or bacterial genes into tumor cells, which causes the ability to apply non-toxic prodrugs into cytotoxic drugs or express toxic gene expression products to kill tumor cells without affecting the normal cells. A main characteristic of suicide gene therapy is its bystander effect, which spreads to the transfected tumor cells after treatment and kills untransfected tumor cells, and the degeneration of distant tumor cells or tumor lesions is observed, further expands the anti-tumor effect of suicide gene. The HSV thymidine kinase (HSV-TK)/ganciclovir (GCV) prodrug system and cytosine deaminase (CD)/5-fluorocytosine (5-FC) are the widely studied suicide gene systems [77]. This enzyme/prodrug combination could promote cell death, not only in recipient cells, but also in neighboring cells, to kill tumor cells through the so-called “bystander effect” [78, 79] constructed CRAd (Ad-ETK) expressing E1A and HSV-TK genes and confirmed that Ad-ETK enhanced OV therapy for HCC [80]. Another widely studied suicide gene system is derived from CD. This enzyme is not expressed in mammalian cells, and it can transform 5-FC, a relatively non-toxic prodrug, into 5-fluorouracil (5-FU), an anticancer drug with high activity and toxicity. In addition, the CD/5-FC system did not cause any severe adverse reactions [81]. The selective killing effect of Ad-mediated CD combined with the TK suicide gene system on HCC cells in vitro and in vivo was studied by constructing the double suicide gene system AD-VEGFp-CDglyTK containing vascular endothelial growth factor promoter (VEGFp), thereby confirming that the combined suicide gene system of TK/GCV and CD/5-FC driven by VEGFp can effectively inhibit the growth of HCC cells, which may provide a potential therapeutic strategy for the treatment of HCC [82].

### Anti-angiogenesis strategy

The generation of tumor vascular system is an essential part of TME, and blood vessels are an important way for tumor growth and metabolism, which can provide nutrients, growth substances and sufficient oxygen for tumor cells to grow, export their metabolic substances [83]. In 1971, Folkman first proposed that the growth and infiltration of tumors depend on the formation of tumor neovascularization, and this theory became the focus in tumor research [84]. Angiogenesis is regulated by a variety of signal transduction pathways of growth factors and cytokine receptors by several angiogenic factors, such as VEGF, basic fibroblast growth factor, platelet-derived growth factor, and anti-angiogenesis factor [85]. In solid tumors, excessive abnormal angiogenesis plays a key role in tumor progression. This process is that the imbalance of pro- and anti-angiogenic factors caused by VEGF overexpression under the tissue hypoxia [86]. VEGF (now referred to as VEGF-A) is a member of protein families, and VEGF-A plays a major role in regulating angiogenesis and diseases [87]. VEGF acts through its homologous TK receptor and some co-receptors. VEGF mRNA is over-expressed in most human tumors and correlates with invasiveness, vascular density, metastasis, recurrence and prognosis [87]; in hypoxia, the expression of VEGF is more active[86]. At present, anti-VEGFA drugs have been used in the clinical treatment of cancer. Bevacizumab is a humanized anti-VEGF monoclonal antibody, which can inhibit tumor angiogenesis by binding with VEGFA and inhibiting its binding with vascular endothelial growth factor receptor (VEGFR)-2. It is the first anti-angiogenesis agent approved by FDA. HCC is a tumor with abundant blood vessels. VEGF/VEGFR signal transduction is closely related to the growth, progression, and metastasis of HCC. Angiogenesis plays a significant role in its development and progression [88]. Huang et al. developed a low-molecular-weight chitosan (LMWC) by constructing the LMWC/VEGF short hairpin RNA (shRNA) complex, and its therapeutic effect was confirmed in ectopic and orthotopic liver cancer models. The drug showed higher efficiency in inhibiting tumor VEGF expression, thereby reducing tumor vascular density and inhibiting tumor growth [89]. Yoo et al. constructed the Ad-based shRNA expression system (Ad-DealtaB7-shVEGF), which can reduce VEGF expression and induce an anti-angiogenesis effect on liver cancer [90]. Angiostatin is a specific angiogenesis inhibitor produced by tumors, and inhibiting the growth of primary and metastatic tumors by blocking tumor angiogenesis has been proven by many different types of solid tumors in animal models. Schmitz et al. constructed the rAd vector of the angiostatin-like molecule (ADK1-3) injected intravenously into athymic mice with subcutaneous HCC, thereby inhibiting the tumor growth [91].

Notably, the traditional anti-angiogenesis strategy effectively promotes drug resistance and metastasis. Some scholars have suggested that anti-angiogenic therapy can correct the structural and functional defects of tumor blood vessels, and this process is known as “blood vessel standardization”. Striking a delicate balance between normalization and excessive vasoconstriction is necessary, and the requirements of dosage selection and administration of antiangiogenic drugs are emphasized. The combination of radiotherapy, chemotherapy, and immunotherapy in the “blood vessel standardization time window” of anti-angiogenic therapy can achieve a better therapeutic effect [83, 92].

## Challenges and solutions of combination therapy with OVs

Combination therapy with OVs is an effective cancer treatment. Therefore, addressing the inefficiency of single-gene therapy, the balance between target gene expression regulation and viral replication, and a series of administration routes is necessary to obtain more safe and effective treatment effects.

### Inefficiency of single-gene therapy

Based on the in-depth study of gene therapy in laboratory and clinical experiments, more targets for tumor gene therapy are revealed, and people are not satisfied with the results of single-gene therapy. In addition, the expression of a single transgene may be insufficient to eradicate tumors, particularly in the late diagnosis of disease. Therefore, multimodal therapy with one or more transgenes must be considered to ensure the success of therapy [93]. Combined gene therapy has improved the therapeutic effect and addressed the shortcomings of single-gene therapy, which has also been carried out in the treatment of liver cancer and achieved effective progress. Galal et al. confirmed that systemic therapy with an OAd inhibitor of growth 4 (ING4) and OAd-TRAIL elicited a more eradicative effect on the orthotopic mice model of human HCC than monotherapy, without apparent overlapping toxicity [94]. Liu et al. explored IL-24 and TRAIL expressed by Ad-AFP-D55-IL-24 and Ad-AFP-D55-TRAIL,which induced apoptosis through Caspase-8 and Caspase-9 signaling pathways, inhibited HCC cell growth, and this combination increased animal survival by inhibiting tumor growth in Huh-7 cell xenograft mice, showing a strong antitumor effect in vivo [95].

In general, tumors tend to have many genetic alterations and intratumoral heterogeneity, moreover, metastatic tumors usually have new mutations. Distinguishing driver mutations from subsequent passenger mutations based on the ability to induce cellular transformation from a large number of genetic mutations in cancer cells may not be feasible, which poses difficulties for single-gene therapy and even gene therapy for cancer, and advances in computational biology allow us to analyze the vast amount of data generated by current cancer genomics projects and predict genetic mutations, genomes, and pathways that drive tumorigenesis [96]. The development of new technologies has also provided convenience to gene therapy for precision therapy, such as single-cell sequencing (SCS) as an emerging high-throughput technology to explore genomics, transcriptomics, and epigenetics at the single-cell level. SCS has gradually become an effective clinical tool to explore tumor metastasis mechanisms and formulate therapeutic strategies, which can be used to identify metastasis-related therapeutic targets, and it provides insight into the distribution of tumor cell subsets and gene expression differences between primary and metastatic tumors [97]. The identification and sequencing of circulating tumor cells (CTC) and cell clusters can identify cell biology expressing candidate genes known to be associated with cancer. Chen et al. used a simple double-filtration method to collect CTC and cell clusters of HCC from patients, and single-cell RNA sequencing found that some of these cells and clusters expressed genes involved in cancer biology, including CSCs and epithelial-mesenchymal transition (EMT) markers, which ensures cancer gene therapy [98]. Furthermore, combined gene therapy can prevent single-gene therapy from being resistant or losing efficacy during gene mutation.

### Application of biological regulation mechanism in liver cancer research

Gene technology is developing rapidly, among which the ability to control the expression level of genes or shRNA in vitro and in vivo is an essential tool to study gene expression timing or dosing. Research on the regulation of gene expression has been involved in various basic and applied biological research fields, including functional genomics, tissue engineering, gene therapy, and biopharmaceuticals. The early inducible gene expression technology primarily relies on endogenous regulatory elements, and its main disadvantage is pleiotropy, which leads to multiple interferences from induction/induction and host regulatory mechanism network. In minimizing or eliminating interferences, various exogenous regulatory systems appear. These exogenous effector molecules can quantitatively and temporally control gene expression in eukaryotic cells [99]. The tetracycline-regulated gene expression system (Tet system) can control the expression of target genes quantitatively and temporally, and it is widely used to control gene expression in eukaryotic cells and organisms, including mammals and insects [100]. The system is based on Tet repressor protein (TetR) and Tet operon (TetO) DNA elements, which control the regulatory elements of tetracycline resistance operon. The binding of Tet or Tet-derivative such as doxycycline (Dox) triggers the conformational change of condensation, which prevents binding with TetO. Based on this principle, two kinds of Tet systems have been developed: Tet-off and Tet-on systems [95]. In 1992, Gossen et al. produced a mixed transactivator (TTA) by combining TetR with the C-terminal domain of HSV VP16, which stimulated the smallest promoter fused with the TetO sequence and established the Tet-off system [101]. Later, they established a reverse system, namely, the Tet-on system: the Tet system was established by using rTTA instead of TTA, which was invalid without Tet, and rTTA was combined with TetO only when Tet or Dox existed, which allows the transgene expression to be induced in a dose-dependent manner [101]. At present, the Tet system is widely used in tumor gene therapy. The transgenic expression can be switched in vivo in the liver, and its delivery is allowed, thereby improving the curative effect of treating gene transfer and limiting toxicity [102]. Fechner et al. constructed a bidirectional expression cassette of OVs Ad.418, which was inserted into the E1 region of the Ad genome. The viral production of Ad.418 progeny is significantly higher in the presence of Dox than in the absence of Dox, and it can kill tumor cells in the presence of Dox, whereas tumor cells are completely unaffected in the absence of Dox [103].

### Selection of cell carriers for intravenous administration of gene therapy

Most studies on tumor gene therapy adopt local intratumoral injection. However, intratumoral injection leads to the uneven distribution of drugs in the tumor, and avoiding systemic leakage is difficult, leading to external transduction, particularly in normal cells scattered in the tumor site, which can also be discharged from the injected tumor site to the circulation and can affect other normal organs/tissues. Intratumoral injection is not feasible in cases with multiple tumor foci. Intravenous administration can solve such problems and can be administered repeatedly than intratumoral administration of some deep tumors, and it is more convenient. However, the intravenous administration of viral vector used for in vivo therapy could cause systemic viral infection and a greater risk of far-reaching spread and metastasis. Many studies of OVs have attempted a variety of carrier tools to improve the delivery of viruses in vivo after intravenous administration, including stem cells, nanoparticles, hydrogels, etc. Moreover, the immune system, blood components, and settled macrophages may be at risk for virus neutralization; thus, virus delivery from vein to tumor must be addressed [104]. Cytokine-induced killer (CIK) cells can identify tumors through related receptors without damaging normal cells. Dai et al. used CIK cells to inject KGHV500 intravenously, showing its anti-tumor effect and safety [105]. MSCs are non-hematopoietic stem cells, which can self-renew, expand in vitro, easily separate, and localize injured tissues, inflammatory sites, and tumors. Therefore, they can be used as potential carriers of anti-tumor genes to treat tumors, and their sources are abundant. MSCs can be isolated from various tissue types, including the bone marrow, umbilical cord blood, adipose tissue, placenta, amniotic fluid, and skin, which can be used as effective cell carriers for tumor gene therapy [106, 107]. The MSCs used as carriers of OV can improve the clinical efficacy of anti-tumor viral therapy by driving Ad to the tumor and recruiting T cells. Yoon et al. confirmed that in MSCs carrying OV, the OV could replicate well, and the protective function of MSCs can increase and promote the circulation of viral particles in the blood. Its tumor-homing tendency can improve the accumulation of tumor-specific viruses, deliver the virus to tumors, and reduce the potential risk of intravenous injection of naked virions. MSC-mediated OV vector can enhance the anti-HCC effect [108]. Research on MSCs carrying OV in an HCC model in situ constructed by Hep3B cells also confirmed that MSCs as cell carriers could enhance the anti-tumor effect of OV, indicating that oAd-MSC therapy can be a potential treatment measure [109]. In addition, neural stem cells can also be used as carriers, which could deliver OVs for cancer therapy, and this strategy was feasible and safe [110]. Table 5 shows different virus delivery systems and summary their advantages and disadvantages.

**Table 5:** Different virus delivery systems and their advantages and disadvantages

Delivery system	Type of OVs	Advantage	Disadvantage
hMSCs	AdV	The higher viral copies can be delivered, and increase the circulation of viral particles	Poor uptake of OVs by hMSCs
NSCs	CRAd	NSCs promote CRAd penetration of the blood–brain barrier, and with robust tumor tropism	Poor infection of mouse tissues by human AdV
Nanoparticle	AdV, MV, NDV	Nanoparticles have a high enhanced permeability and retention (EPR) effect, which is easier to penetrate into tumor tissues and the retention time is longer	Nanoparticles release drug effector substances that are easily degraded
Hydrogel	CRAd	The stronger cytotoxicity to cancer cells and long-term antitumor therapeutic effects	Hydrogel may lead to increased toxicity and also damage surrounding cells

## Combination therapy strategies for liver cancer

Many researches have been conducted on gene therapy for liver cancer, all of which have achieved considerable curative effect. Studies have confirmed that the effect of combined gene therapy is better than that of single-gene therapy, and the OV with an oncolytic effect is selected as the delivery carrier [93]. Given the limitation of local intratumoral injection, intravenous injection can be selected. In addition, MSCs with tumor-homing function can be used in tumor gene therapy to overcome the influence of Ad vector on normal tissue cells and enhance its specificity to tumor cells. By adding an exogenous regulatory mechanism of the Tet system, the expression level of the tumor-suppressor gene carried by the viral vector can be increased, and the potential replication and reproduction of the virus at the tumor site can be reduced, which may cause damage to normal tissues and organs. Therefore, the OV vector of combined genes regulated by the Tet system was constructed. The research idea of using chemotactic MSCs as carrier to treat liver cancer by tail vein injection has potential feasibility. In the study of glioma, Zhang et al. established an OV carrying IL-24/Endostatin and used MSCs as the carrier to study glioma through tail vein injection, indicating that this therapeutic system can effectively inhibit the proliferation of glioma cells in vitro and the growth of the subcutaneous glioma tumor model in vivo [93]. The in situ model of human glioma is a limitation of this study. In the study of glioma in vivo, the entry of many therapeutic drugs into the central nervous system is almost restricted because of the presence of the blood–brain barrier (BBB) [111], whereas MSCs can pass through the BBB. Moreover, the systemic delivery of stem-cell-based therapeutic agents is a feasible and efficient treatment method that allows the non-invasive and repeated application to target malignant glioma [112]. The liver has dual blood supply channels; thus, the blood flow is abundant, and BBB has no effect. Extensive research shows that intravenous injection can effectively accumulate Ad in the liver, and MSCs as the systemic carrier of OAd can improve the accumulation of OAd in tumors and reduce hepatotoxicity and adverse reactions of blood circulation [109]. Therefore, intravenous or arterial administration may achieve a higher, more effective, and safe drug treatment concentration in liver cancer. This method may produce better effects in liver cancer, and it has greater clinical therapeutic significance for the treatment of liver cancer, which needs further exploration.

The occurrence and development of cancer are complex processes involving multiple biological pathways, such as the excessive proliferation of tumor cells, resistance to apoptosis, evasion of the immune system, angiogenesis, survival, and colonization of distant tissues, and these changes are intricate. The interaction network is primarily due to the mutation of tumor genes, which have a high degree of heterogeneity; however, the molecular genetic characteristics of the same tumor are different. A single treatment modality has limitations, and obtaining satisfactory treatment results is difficult. At present, extensive molecular and immunological evidence has demonstrated that HCC is a heterogeneous cancer with different etiologies, mutations, and immune microenvironments [113], and a combination of multiple approaches is necessary. In recent years, research on HCC immunotherapy has grown significantly and changed the treatment paradigm for cancer [114]. Thus, it becomes an important approach for the treatment of HCC, in combination with vaccines, OVs, and conventional therapy for patients with different stages [115, 116]. Immune checkpoint blockade and adoptive cell therapy are effective means of immunotherapy [115]. Immune checkpoint inhibitors are monoclonal antibodies (mAbs) that selectively block inhibitory immune checkpoints such as programmed death 1 (PD-1), programmed death ligand 1 (PD-L1), and cytotoxic T lymphocyte antigen 4 (CTLA-4), thereby enhancing T cell-mediated antitumor immune responses [116]. Among them, PD-1 signaling is often hijacked by cancer cells to evade immune surveillance, and blocking PD-1 or its ligand PD-L1 has been approved for the treatment of various solid and hematological malignancies [117]. Over the past decade, advances in immunotherapy combined with the improved virus engineering have led to new therapeutic ideas for OV therapy. Combining OV therapy and immunotherapy could enhance cancer outcomes compared with their monotherapies [118, 119]. By constructing an AdV (AdC68-spE1A-aPD-1 expressing aPD-1), the oncolytic ability of AdV is retained, whereas aPD-1 is efficiently secreted from infected tumor cells, and it specifically binds to PD-1 protein. It exhibits tumor-suppressive effects, including liver cancer cells in vitro and in vivo [118]. Kanaya et al. designed a fusion protein containing PD-1 and PVR and inserted the corresponding expression fragment into the genome of AdV to construct Ad5sPD1PVR. Studies in HCC cell lines confirmed that Ad5sPD1PVR can significantly enhance the antitumor efficacy mediated by CD8 + T cells, and it has a long-term tumor-specific immune monitoring effect [119]. Chimeric antigen receptor T-cell (CAR-T) therapy is a potential and rapidly developing approach in treating hematological malignancies, but its application in solid tumors has been limited because of the heterogeneous expression of antigens and induction of immunosuppression in the TME. The use of CAR-T cells with OVs can enhance the efficacy of CAR-T cell therapy in destroying solid tumors, increase the permeability of tumor cells to T cells, and reduce the interference effect of tumor cells, which can also improve the TME by producing type I INF to transform “cold tumor” into “hot tumor” and promote the infiltration, activation, and proliferation of CAR-T cells. Preclinical studies and various animal models of cancer have demonstrated that combination therapy is superior to single gene therapy, and it can improve cancer therapy, particularly for solid tumors [120, 121]. Researchers loaded reovirus/vesicular stomatitis virus with CAR-T cells. After CAR-T cells delivered OVs to solid tumor mouse models of melanoma and glioma, the virus infiltrated tumor cells for replication and then ruptured tumor cells and elicited an effective immune response, indicating that lysis tumor virus greatly enhances tumor efficacy of CAR-T cells in mouse models of melanoma and glioma and improves the survival of mouse [122]. Therefore, CAR-T combined with OVs could treat many solid tumors, including liver cancer.

Moreover, recent studies of OVs-based combination therapy in liver cancer have been widely studied. Liu et al. explored the combination of a ferroptosis activator with an oncolytic vaccinia virus in tumor models, including hepatocellular carcinoma and colon cancer models, either erastin or oncolytic vaccinia virus (OVV) inhibited tumor growth, but a combination of the two yielded the best therapeutic effects, as indicated by inhibited tumor growth or regression and longer host survival [123]. OV therapy is expected to revolutionize the treatment of liver cancer. Li et al. developed a recombinant oncolytic influenza virus carrying GV1001 triggers an antitumor immune response, which suggested that oncolytic influenza virus carrying GV1001 was a promising immunotherapy in patients with HCC [124].

## Summary and prospects

**Fig. 2:**
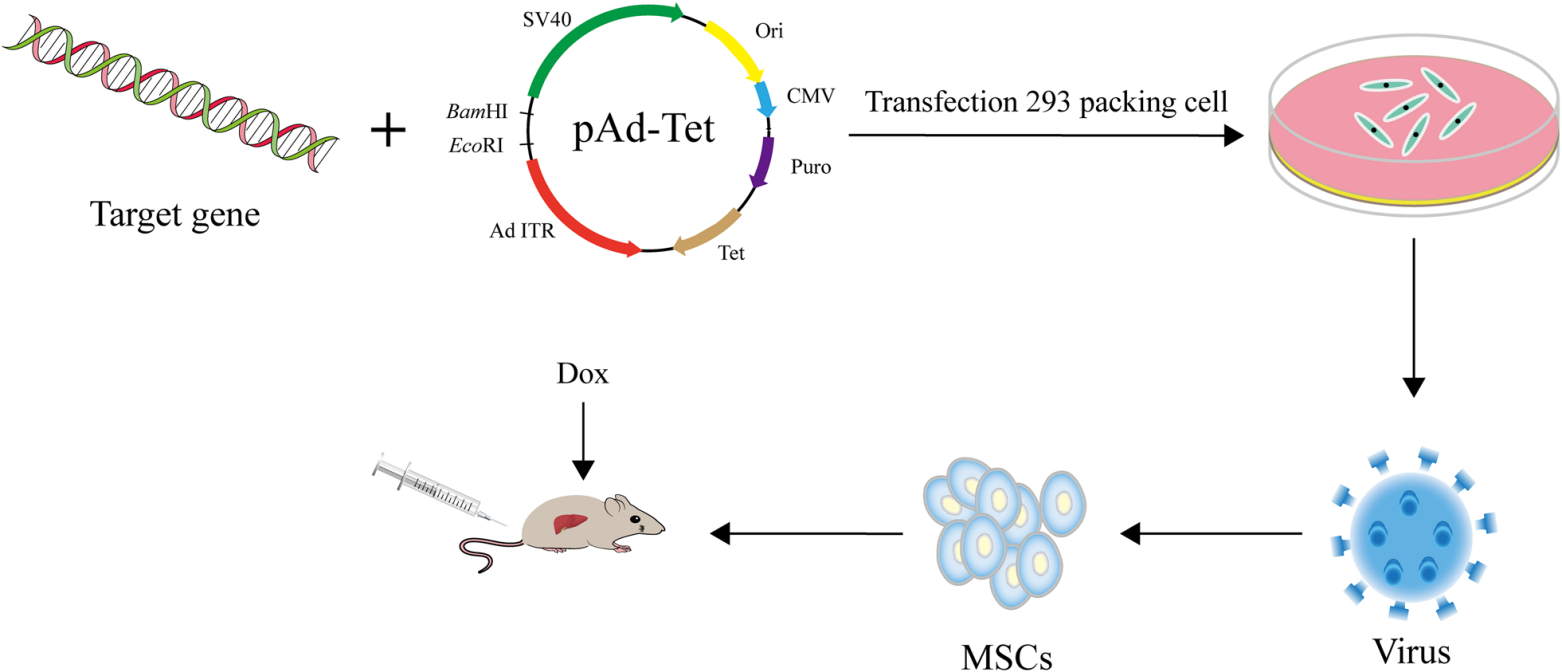
Gene therapy strategies of mesenchymal stem cells loaded with oncolytic adenovirus carrying target genes regulated by a tetracycline system. MSCs loaded with OVs carrying target genes are injected into the tail vein, and viral replication is regulated by a tetracycline system, which can be a useful strategy for the treatment of liver cancer in vivo

At present, OV has emerged as a powerful therapeutic approach in cancer treatment. Genetically engineered OVs have been widely studied as the primary gene therapy vector. Many engineered OVs were used for the treatment of liver cancer, including HSV-1, NDV, MV, poxvirus, and AdV. Combined with previous treatment measures, an improved strategy based on engineered OVs was summarized following the target gene, regulation mode, and drug administration, and the engineered OAd carrying therapeutic genes controlled by the Tet system was constructed. Moreover, MSCs were used to carry the virus, and the treatment of liver cancer was studied by tail vein injection (Fig. 2). Based on previous research, the use of MSCs is feasible. Although gene therapy has great application potential to treat cancer, which is primarily carried out by in vitro tumor cells, animal models of liver cancer, and pre-clinical trials, its clinical application still has limitations. Scientists should consider its safety and effectiveness. Comprehensively understanding the molecular genetic mechanism of carcinogenesis and rapidly developing a gene delivery technology, the discovery of advanced molecular monitoring and TME is crucial, and the establishment of animal models is necessary to explore specific molecular traits and tumor phenotype of liver cancer [125]. Considerable studies have proven that OV therapy combined with other treatment methods, particularly immunotherapy, can significantly enhance the anti-tumor effect, opening up a new way for tumor therapy. In recent years, preclinical and clinical trials of OVs-based combination therapy for liver cancer have been studied, and the results show that the oncolytic and immune-stimulating effects of OVs are more effective when combined with other therapies. Thus, with the development of genetic technology, OV is a new potential therapeutic modality that harnesses virus biology and host interactions to treat liver cancer, which will remarkably increase its potential clinical application.

## Data Availability

Not applicable.
